# Effects of psychosocial and socio‐environmental factors on anxiety disorder among adolescents in Bangladesh

**DOI:** 10.1002/brb3.1899

**Published:** 2020-10-21

**Authors:** Md. Mostaured Ali Khan, Md. Nuruzzaman Khan

**Affiliations:** ^1^ Department of Population Science and Human Resource Development University of Rajshahi Rajshahi 6205 Bangladesh; ^2^ Practical Action MEL and Research Dhanmondi Dhaka 1205 Bangladesh; ^3^ Research Centre for Generational Health and Ageing School of Public Health and Medicine Faculty of Health and Medicine The University of Newcastle Callaghan NSW Australia; ^4^ Department of Population Science Jatiya Kabi Kazi Nazrul Islam University Mymensingh 2220 Bangladesh

**Keywords:** adolescents, adverse childhood experiences (ACE), anxiety disorder, Bangladesh

## Abstract

**Background:**

Anxiety is a common psychiatric disorder among adolescents in developing countries. This study aimed to examine the risk factors of anxiety disorder and the adverse consequences of anxiety disorder among adolescents in Bangladesh.

**Methods:**

A total of 2,989 adolescent data of the 2014 Global School‐based Student Health Survey (GSHS), Bangladesh, was analyzed WHO. The prevalence of anxiety disorder across psychosocial and socio‐environmental factors was calculated as descriptive statistics, whereas their effects on occurring anxiety disorder were determined using the unadjusted and adjusted multivariable binary logistic regression model. The consequences of anxiety disorder were also determined using the multivariable binary logistic regression model.

**Results:**

The prevalence of anxiety disorder was 4.7%, which was found higher among female than their male counterparts. The psychosocial factors were found associated with the anxiety disorder are loneliness (AOR: 2.26, 95% CI: 1.08–4.72), being bullied (AOR: 6.00, 95% CI: 3.14–11.47), and physical abuse (AOR: 2.12, 95% CI: 1.07–4.21). Moreover, poor understandings with parents (AOR: 1.75, 95% CI: 1.02–3.01) and lack of peer support (AOR: 2.23, 95% CI: 1.18–4.23) were the socio‐environmental factors that found associated with the anxiety disorder. Subgroup analysis across gender found these associations were consistent for adolescent male and female. Moreover, the likelihood increased with the increasing number of the adverse psychosocial, or socio‐environmental factors.

**Conclusions:**

Around 5% of school‐going adolescents in Bangladesh reported anxiety associated with the exposure of single or multiple adverse psychosocial and socio‐environmental factors including bullying and physical abuse. Early screening and interventions are essential, targeted to adolescent at risk, which could reduce the rate of anxiety disorder among adolescent in Bangladesh.

## Graphical abstract

The current study reported around 5% of anxiety disorder among adolescent in Bangladesh and it significantly increases the tendencies of adolescent’s suicidal behaviour (ideation, plans and attempts) and alcohol and drug abuse tendencies. Several single and multiple adverse psychosocial and socio‐environmental experiences are accelerating the risk.

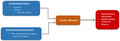

## 1 INTRODUCTION

Understanding adolescent psychological problems are turning into a growing need for the global health and safety concern, of which anxiety disorder is very crucial. It includes a set of mental sicknesses that characterized by excessive fear (that could be unrealistic), worry, obsessive thoughts, and sleep disturbance due to worry or fear (Joyce‐Beaulieu & Sulkowski, 2016; Parekh, 2019). The 2017 Global Burden of Disease Study found around 284 million (3.8%) people is suffering from different types of anxiety disorder worldwide, with a 32.3% increase from 1990 to 2017 (James et al., 2018). Around 23% of these population with the anxiety disorder live in South‐East Asian countries (WHO, 2017a, 2017b). This prevalence is even higher among adolescent at around 6% prevalence worldwide which further varies across regions (WHO, 2017a, 2017b) and gender (prevalence is higher among female than male adolescent) (James et al., 2018; WHO, 2017a, 2017b).

There are several forms of anxiety disorder reported worldwide. These are generalized anxiety disorder (GAD), social anxiety disorder, panic disorder, obsessive‐compulsive disorder (OCD), phobias, and post‐traumatic stress disorder (PTSD). Their symptoms are different and could be ranged from mild to severe (WHO, 2017a, 2017b), however, each form affects adolescent education and social and family lives substantially (Creswell et al., 2014). Consequently, it increases the risks of lower self‐esteem, depression, tobacco use, illicit drug dependence, and suicidal behavior (Essau et al., 2014; Lawrence et al., 2017; Lee & Hankin, 2009). These are challenging for adolescent healthy life and development; extensive work and continued efforts are therefore important to identify the factors associated with anxiety disorders. This could help the policymakers and planners to take evidence‐based policies targeting to reduce anxiety disorder among adolescent.

A limited number of studies have been conducted to date aiming to identify the etiology and determinants of anxiety disorder; however, these were mainly for adulthood (aged 21 years or more). They found a gender difference in the occurrence of anxiety disorder, girls were more likely to reported anxiety disorder than boys (Essau et al., 2014). Other important determinants of anxiety disorder identified were different intrinsic, familial, and social environmental factors. These were lack of family support, parental and school aversiveness, poor understanding with parents, interparental conflicts (Cardamone‐Breen et al., 2017), parents with a mental disorder (Ghandour et al., 2019), low peer relationship, lower number of a close friend, and relational victimization (Pickering et al., 2019; Siegel et al., 2009). Excessive stress, loneliness (both social and emotional), and experience of peer victimization or bullying (physical, verbal, or psychological) were also found to be associated with the increased risk of developing adolescent anxiety disorder (Acquah et al., 2016; Alisic et al., 2014; Swearer & Hymel, 2015). Abuse, both physical and mental, was also found as a significant determinant of the anxiety disorder (Rehan et al., 2017). Moreover, the excessive use of digital and social media, including Facebook, Twitter, etc., was found associated with increased risk of the anxiety disorder (Hoge et al., 2017), mainly due to lack of emotional regulation skills and social attachments (Hoge et al., 2017).

Around 7 million people, represents around 4.4% of the total population in Bangladesh, faces different forms of the anxiety disorder (WHO, 2017b). The World Health Organization in 2017 reported this prevalence was 5% among adolescent, a group that represents around 10.2% of the total Bangladeshi population (WHO, 2017a, 2017b). However, the factors associated with such higher prevalence of anxiety disorder among adolescent in Bangladesh are mostly unknown. There are relatively few studies on this aspect that have been conducted in Bangladesh identified some specific factors (e.g., physical violence, poor partner relationship, etc.) of anxiety in the antepartum period (Nasreen et al., 2010, 2011). A few other studies identified factors associated with mental disorders and their associated comorbidities (Hossain et al., 2014). Moreover, a recent study found that around 11% of adults in Bangladesh were suffered from anxiety disorder (Sadiq et al., 2019). Importantly, these studies were also equivocal concerning the prevalence of the anxiety disorder and its associated factors. Moreover, global level studies found a different set of factors associated with the anxiety disorder (e.g., bullying, poor peer support, etc.) than the factors reported in these Bangladeshi studies (Bandelow & Michaelis, 2015; James et al., 2018; Lee et al., 2007). Moreover, so far no study has been conducted to assess the risk of anxiety disorder in case of multiple adverse events, such as a person faces more than one adverse psychosocial or socio‐environmental risk factors at a time, though this scenario is common in Bangladesh.

This study is therefore conducted to identify the prevalence of anxiety disorder among adolescent of Bangladesh and its associated individual, psychosocial or socio‐environmental risk factors. Effects of anxiety disorder on different adverse consequences among adolescents in Bangladesh are also determined.

## 2 DATA AND METHODOLOGY

### 2.1 Sample design

Data from the 2014 Bangladesh Global School‐based Student Health Survey (GSHS) were analyzed (2018). The World Health Organization (WHO) conducts this survey in 44 developing countries whereas the Centers for Disease Control (CDC), USA provided financial and technical supports. The primary target group was adolescent. The survey used a two‐stage stratified cluster sampling technique. Schools were selected at the first stage through probability proportional to enrollment size, whereas classes in each selected school were selected in the second stage through random sampling. Finally, students studying in the selected classes (class 7–10, age 11–18) were selected and interviewed using a conventional school‐based questionnaire module. Every student in the selected classes was eligible to participate in the survey, whereas they were completed a self‐administered questionnaire. Broad description regarding this survey and sampling procedure can be found in the WHO’s webpage (https://www.who.int/ncds/surveillance/gshs/en/) (WHO, 2018).

### 2.2 Sample size

A total of 2,978 adolescent responds to the question of anxiety disorder was included in this study. This sample extracted from 2,989 adolescent interviewed in the survey. The interviewed adolescent was asked several questions related to dietary behaviors, mental health, physical activity, sexual behaviors, tobacco, alcohol, and drug use.

### 2.3 Outcome variables

Anxiety disorder among adolescent was our primary outcome of interest. During survey, each interviewed adolescent was asked, *“During the past 12 months, how often have you been so worried or panic about something, or you could not sleep at night due to tension or panic?”*. Responses were recorded in 5 points Likert scale: never (1), rarely (2), sometimes (3), most of the time (4), and always (5). We recoded these responses to create dichotomous anxiety disorder variable: Yes [1] versus No [0]. Respondents responded either most of the time (4) and always (5) were recorded as “Yes” to anxiety disorder, and otherwise considered as “No” response to anxiety disorder. The classification was done based on previous research (Asante et al., 2017; Randall et al., 2014).

We have also selected five adverse outcome variables available in the dataset to see their associations with the anxiety disorder. These were suicide ideation (yes, no), suicide plan (yes, no), suicide attempts (yes, no), alcohol use (yes, no), and drug use (yes, no). Information on suicide plan, ideation, and attempt was collected by asking the respondents “*how many suicide plans, ideations, or attempts they had taken in past 12 months*?”. Response options were never (1), rarely (2), sometimes (3), most of the time (4), and always (5). Responses recorded were recoded positively to use in this study if respondents’ reported any suicide plan, ideation, or attempt, otherwise recoded as negative response to the respective questions. Information on alcohol use was collected by asking the respondents “*how many times drank alcohol in past 30 days*? At least one drink of alcohol during this period was considered positive response to alcohol use. Similarly, adolescents drug use information was collected by asking the respondents about how many times they had taken any drugs (cannabis, amphetamine, etc.) in lifetime and recoded as “1” if they ever used, otherwise “0”.

### 2.4 Explanatory variables

A set of socio‐demographic, psychosocial, and socio‐environmental factors were used as the explanatory variables, which were selected in two stages. A list of explanatory variables was first generated by reviewing the relevant literature (Arat & Wong, 2016; Asante et al., 2017; Khan et al., 2020; Pickering et al., 2019; Randall et al., 2014; Swearer & Hymel, 2015). The forward regression model was then used to select the explanatory variables for this study. We also checked the multicollinearity of the selected variables using variance inflation factor (VIF) and dropped the relevant variable if evidence of multicollinearity was found (the VIF values were <2 in all variables included in this study). The complete list of explanatory variables, their categories, and coding are presented in Table 1.

**Table 1:** The complete list of independent variables

Variables	Category	Variable type
Age	11–17 years (coded categorically)	Demographic variables
Gender	1 = Male	
	2 = female	
School grade	1 = Class VII	
	2 = Class VIII	
	3 = Class IX	
	4 = Class X	
Food insecurity (useful proxy of socioeconomic status)^a^	0, No = Never/rarely/sometimes	
	1, Yes = Most of the time/always	
Loneliness^b^	0, No = Never/rarely/sometimes	Psychosocial risk factors
	1, Yes = Most of the time/always	
Bullied^a^	0, No = Never	
	1, Yes = One or more days	
No close friends	0, No = Have at least one close friend	
	1, Yes = No close friends	
Ever sexual intercourse	0, No	
	1, Yes	
Smoke cigarette or other tobacco^a^ (including marijuana)	0, No	
	1, Yes = One or more days	
Physically abused^b^	0, No	
	1, Yes = One or more times	
Parents rarely check homework^a^	0, No = Never/rarely/sometimes 1, Yes = Most of the time/always	Socio‐environmental factors
Poor understanding with parents^a^		
Poor parental monitoring^a^		
Lack of positive peer support^a^		
Truancy^a^		
Parental tobacco or drug abuse	0, No	
	1, Yes	

^a^ In past 30 days.

^b^ In the past 12 months.

Moreover, of the explanatory variables were selected finally for this study, we have classified relevant variables in two broad categories: psychosocial factors and socio‐environmental factors. We then count the number of adverse responses under each category to calculate Multiple Adverse Experience (MAE) scores. For instance, if a person had three adverse experiences then the MAE score was 3. We followed the Adverse Child Experience (ACE) score generation procedure to calculate MAE score. The ACE score generation procedure was developed and designed by Kaiser Permanente in collaboration with the Centers for Disease Control and Prevention, conducted from 1995 to 1997 (Felitti et al., 1998).

### 2.5 Statistical analysis

Unadjusted and age‐adjusted prevalence of anxiety disorder were calculated across psychosocial and socio‐environmental factors. Age‐adjusted prevalence was calculated using the age‐standardized weights derived from the national population and housing census of Bangladesh 2011. The association between the anxiety disorder and different individual, psychosocial and socio‐environmental factors was determined using a multivariable binary logistic regression model. Both unadjusted and adjusted model were considered. The anxiety disorder variable with a specific variable was considered in the unadjusted model, whereas anxiety disorder variable was considered with all individual, psychosocial and socio‐environmental factors in the adjusted model. A set of five different multivariable binary logistic regression models was also used to the association between anxiety disorder with suicide ideation, suicide plan, suicide attempt, alcohol use, and drug use adjusting with possible confounders. Complex survey design and sampling weights were considered in all analyses. We have done these analyses following imputation of the missing value (if any and not more than 5%) for explanatory variables using logistic regression model (Khan et al., 2020; Sterne et al., 2009). The STATA program version 13.0 *SE* (StataCorp. LP) was used for all analyses.

## 3 RESULTS

### 3.1 Prevalence of anxiety disorder

Table 2 represents the unadjusted and age‐adjusted prevalence of anxiety disorder across individual, psychosocial, and socio‐environmental factors. The unadjusted and age‐adjusted prevalence of adolescent anxiety disorder were 4.7% and 5.0%, respectively. The prevalence was higher among Grade 10 (14.6%) and male (6.6%) students. Prevalence was also found higher among students reported loneliness (17.8% vs. 3.4%), being bullied (13.8% vs. 2.0%), sexual experience (37.0% vs. 3.8%), and smoke cigarette or other tobacco (43.1% vs. 3.4%) than those adolescents who did not experience such adverse factors. The age‐adjusted prevalence of anxiety disorder was found around double among the students whose parents rarely check homework (7.3% vs. 3.4%), who lacked peer support (7.3% vs. 4.2%), and who reported their parents to use tobacco and drugs (10.6% vs. 3.9%) than their counterpart. Moreover, we found a cumulative increase of age‐adjusted prevalence of anxiety disorder with the increase of the number of adverse psychosocial, and socio‐environmental factors.

**Table 2:** Prevalence of anxiety disorder according to different socio‐demographic and adolescent's adverse experiences in Bangladesh: Global School‐Based Health Survey (GSHS), 2014 (*n* = 2,978)

Background characteristics	Prevalence of anxiety in adolescents (%, 95% CI)	
	Unadjusted	Age‐adjusted
Prevalence of anxiety disorder	4.7 (3.6–6.1)	5.0 (2.5–7.5)
Age		
11–12	4.3 (1.3–13.3)	4.3 (0.8–9.3)
13	5.2 (3.4–7.7)	5.1 (3.1–7.2)
14	4.1 (2.7–6.1)	4.1 (2.4–5.7)
15	4.4 (2.9–6.7)	4.4 (2.5–6.2)
16–18	7.1 (2.2–20.8)	7.1 (1.7–15.2)
School grade		
Class VII	4.9 (3.2–7.5)	3.2 (1.1–5.4)
Class VIII	5.0 (3.3–7.5)	4.3 (1.7–8.4)
Class IX	3.8 (2.6–5.4)	2.1 (1.2–2.9)
Class X	5.0 (2.1–11.6)	14.6 (4.4–33.6)
Gender		
Male	4.5 (2.9–6.7)	4.9 (2.4–7.4)
Female	5.2 (3.9–6.8)	6.6 (2.8–10.4)
Food insecurity		
No	4.7 (3.6–6.0)	5.0 (2.8–7.3)
Yes	5.4 (2.6–10.9)	6.2 (3.4–13.2)
Adverse experiences in adolescence		
Psychosocial risk factors		
Loneliness		
No	2.9 (2.1–3.9)	3.4 (1.2–5.6)
Yes	19.2 (12.9–27.6)	17.8 (7.2–28.3)
Bullied		
No	3.0 (2.2–4.2)	2.0 (1.3–2.7)
Yes	9.9 (6.8–14.3)	13.8 (7.0–20.4)
No close friends		
No	4.6 (3.4–6.1)	5.2 (2.5–7.9)
Yes	6.2 (2.8–12.9)	3.6 (0.8–6.2)
Ever sexual intercourse		
No	4.6 (3.4–6.1)	3.8 (2.0–5.7)
Yes	5.5 (2.8–10.7)	37.0 (35.6–38.5)
Smoke cigarettes or other tobacco		
No	4.2 (3.1–5.5)	3.4 (2.0–4.7)
Yes	9.9 (5.2–17.8)	43.1 (37.5–48.6)
Physical abused		
No	2.2 (1.3–3.8)	3.2 (1.8–8.7)
Yes	6.1 (4.5–8.2)	4.8 (2.2–7.4)
No. of psychosocial factors		
0	2.1 (1.0–4.0)	2.5 (0.8–5.5)
1	2.8 (1.6–4.8)	1.6 (1.1–2.4)
2	5.7 (3.5–8.9)	5.6 (1.4–9.7)
≥3	14.7 (10.3–20.8)	25.8 (7.7–44.0)
Socio‐environmental factors		
Parental rarely homework check		
No	4.2 (2.6–6.8)	3.4 (0.6–6.2)
Yes	5.3 (4.1–6.8)	7.3 (3.2–11.4)
Poor understanding with parents		
No	3.7 (2.3–5.9)	4.1 (1.0–7.4)
Yes	5.6 (4.2–7.5)	5.9 (3.4–8.1)
Poor parental monitoring		
No	5.3 (3.5–8.1)	6.1 (2.4–9.8)
Yes	4.2 (3.1–5.8)	5.1 (3.5–9.8)
Lack of peer support		
No	3.8 (2.9–5.0)	4.2 (2.2–6.2)
Yes	9.5 (5.4–16.4)	7.3 (3.1–14.2)
Parental tobacco or drug use		
No	3.7 (2.7–5.3)	3.9 (1.9–6.8)
Yes	6.8 (4.4–10.3)	10.6 (3.6–17.7)
Truancy		
No	4.1 (3.1–5.5)	3.6 (1.8–5.3)
Yes	6.1 (3.9–9.3)	9.4 (1.9–18.1)
No. of adverse socio‐environmental factors		
0	1.0 (0.3–2.6)	1.0 (0.7–2.3)
1	3.6 (1.9–6.8)	2.6 (0.9–4.5)
2	5.4 (3.9–7.5)	7.6 (1.3–14.0)
≥3	6.1 (3.3–10.9)	8.9 (6.7–14.9)

Note

All percentages are weighted. Percentages may not total 100.0 because of rounding.

### 3.2 Risk factors of anxiety disorder

The association of adolescent's anxiety disorder with adolescents’ adverse experiences is presented in Table 3. We found a higher odds of anxiety disorder among female adolescents (AOR: 2.06, 95% CI: 1.12–3.77) compared to male adolescent. Students reported loneliness (aOR: 2.26; 95% CI: 1.08–4.72), being bullied (aOR: 6.00; 95% CI: 3.14–11.47), and physically abused (aOR: 2.12; 95% CI: 1.07–4.21) reported higher odds of anxiety disorder than their counterparts. Moreover, poor understandings with parents and lack of peer support appeared to had 1.75 times (95% CI: 1.02–3.01) and 2.23 times (95% CI: 1.18–4.23) higher likelihoods of anxiety disorder, respectively, than their counterparts. Effects of these risk factors are comparatively higher for female adolescents on accelerating the anxiety disorder (Table S1).

**Table 3:** Association between adolescents’ anxiety disorder and adolescent's adverse experiences in Bangladesh: Global School‐Based Health Survey (GSHS), 2014

Risk Factors	Anxiety disorder in adolescents			
	uOR	95% CI	aOR^a^	95% CI
Gender				
Male^(RC)^	1.00		1.00	
Female	1.61^*^	(1.11–2.34)	2.06^*^	(1.12–3.77)
Adverse experiences in adolescence				
Psychosocial risk factors				
Loneliness				
No^(RC)^	1.00		1.00	
Yes	3.54^**^	(2.50–5.01)	2.26^*^	(1.08–4.72)
Bullied				
No^(RC)^	1.00		1.00	
Yes	8.59^**^	(5.96–12.37)	6.00^**^	(3.14–11.47)
No close friends				
No^(RC)^	1.00		1.00	
Yes	1.30	(0.75–2.27)	0.91	(0.38–2.18)
Ever sexual intercourse				
No^(RC)^	1.00		1.00	
Yes	1.04	(0.57–1.91)	0.87	(0.36–2.11)
Smoke cigarettes or other tobacco				
No^(RC)^	1.00		1.00	
Yes	1.56	(0.91–2.69)	1.67	(0.61–4.49)
Physically abused				
No^(RC)^	1.00		1.00	
Yes	3.89^**^	(2.49–6.08)	2.12^*^	(1.07–4.21)
Socio‐environmental factors				
Parental rarely homework check				
No^(RC)^	1.00		1.00	
Yes	1.48^*^	(1.05–2.09)	1.29	(0.71–2.36)
Poor understanding with parents				
No^(RC)^	1.00		1.00	
Yes	1.56^*^	(1.03–2.06)	1.75^*^	(1.02–3.01)
Poor parental monitoring				
No^(RC)^	1.00		1.00	
Yes	1.10	(0.78–1.56)	0.55	(0.33–1.01)
Lack of peer support				
No^(RC)^	1.00		1.00	
Yes	1.83^**^	(1.20–2.78)	2.23^**^	(1.18–4.23)
Parental tobacco or drug use				
No^(RC)^	1.00		1.00	
Yes	1.61^**^	(1.12–2.29)	1.64	(0.86–3.12)
Truancy				
No^(RC)^	1.00		1.00	
Yes	0.84	(0.58–1.21)	1.36	(0.73–2.54)

Note

Values with superscript asterisks * and ** indicate *p* < .05, and *p* < .01, respectively.

Abbreviations: aOR, adjusted odds ratio; CI, confidence interval; RC, Reference category; uOR, unadjusted odds ratio.

^a^ Model was adjusted for individuals’ age, school grade, food insecurity and all the predictors included in this table.

### 3.3 Effect of multiple adverse experiences (MAE)

Table 4 presents the odds of the association of adolescents’ anxiety disorder with psychosocial and socio‐environmental factors. Both the unadjusted and adjusted odds ratios (AOR) were calculated. We found adolescents who experienced at least 2 and ≥3 adverse psychosocial factors had 3.19 times (95% CI: 1.47–6.95), and 9.71 times (95% CI: 4.09–23.06) higher likelihoods of having anxiety disorder, respectively, compared to who did not experience any adverse psychosocial factors. Moreover, relative to those who did not experience any forms of adverse socio‐environmental factors, those who experienced at least 2, and ≥3 adverse socio‐environmental factors had increased likelihood of anxiety disorder by 4.41 times (95% CI: 1.63–11.88), and 5.61 times (95 CI: 1.66–18.96), respectively. There is no difference is observed in case of multiple psychosocial risk factors but multiple socio‐environmental risk factors are affecting more in females compared to male adolescents (Table S2).

**Table 4:** Influence of multiple adverse experiences (MAE) on adolescents’ anxiety disorder

Multiple adverse experiences	Anxiety disorder in adolescents			
	uOR	95% CI	aOR^a^	95% CI
No. of psychosocial risk factors				
0^(RC)^	1.00		1.00	
1	1.35	(0.49–3.69)	1.51	(0.55–4.09)
2	2.88^**^	(1.29–6.41)	3.19^**^	(1.47–6.95)
≥3	8.31^**^	(3.71–18.56)	9.71^**^	(4.09–23.06)
No. of adverse socio‐environmental factors				
0^(RC)^	1.00		1.00	
1	3.62^**^	(1.42–9.21)	3.58	(0.88–8.62)
2	5.49^**^	(1.99–15.14)	4.41^*^	(1.63–11.88)
≥3	6.27^**^	(1.85–21.19)	5.61^**^	(1.66–18.96)

Note

Values with superscript asterisks * and ** indicate *p* < .05, and *p* < .01, respectively.

Abbreviations: aOR, adjusted odds ratio; CI, confidence interval; RC, Reference category; uOR, unadjusted odds ratio.

^a^ Models were adjusted for age, gender, school grade, and food insecurity variable.

### 3.4 Consequences of anxiety disorder

Effects of adolescent anxiety disorder on selected adverse consequences are presented in Table 5. We found a gradual increase in the likelihoods of adverse consequences with the increasing frequency of the anxiety disorder. Higher likelihoods of suicidal ideation (aOR, 4.23, 95% CI, 2.21–8.11), suicidal plan (aOR: 3.93, 95% CI: 2.17–7.09), suicidal attempt (aOR: 6.52, 95% CI: 3.72–11.41), and drug use (aOR: 3.41, 95% CI: 1.32–8.78) were found among the adolescent who reported that they feel anxiety most of the times compared to those who never feel anxiety. Similarly, the likelihood of suicidal ideation, plan, attempt, alcohol and drug use was 12.71 times (95% CI: 4.41–36.60), 5.93 times (95% CI: 2.18–11.22), 8.09 times (95% CI: 2.97–21.98), 25.43 times (95% CI: 7.83–42.58) and 12.69 times (95% CI: 3.74–23.06) higher among the adolescent who reported that they always feel anxiety relative to those who never feel anxiety.

**Table 5:** Effects of adolescent's anxiety disorder on selected adverse consequences: Global School‐Based Health Survey (GSHS), 2014

	Adjusted odds ratio (aOR) (95% CI)				
	Suicide ideation^a^	Suicide plan^a^	Suicide attempts^a^	Alcohol use^b^	Drug use^c^
Feeling anxiety					
Never^(RC)^	1.00	1.00	1.00	1.00	1.00
Rarely	0.35 (0.16–0.75) ^*^	0.37 (0.18–0.74) ^*^	0.65 (0.36–1.17)	1.20 (0.50–2.85)	1.18 (0.57–2.44)
Sometimes	1.25 (0.82–1.89)	1.43 (0.98–2.03)	1.32 (0.89–1.96)	0.98 (0.45–2.12)	0.60 (0.29–1.24)
Most of the time	4.23 (2.21–8.11) ^**^	3.93 (2.17–7.09) ^**^	6.52 (3.72–11.41) ^**^	2.28 (0.64–8.11)	3.41 (1.32–8.78) ^**^
Always	12.71 (4.41–36.60) ^**^	5.93 (2.18–11.22) ^**^	8.09 (2.97–21.98) ^**^	25.43 (7.83–42.58) ^**^	12.69 (3.74–23.06) ^**^

Note

Each of the models were adjusted for individuals’ age, school grade, gender, food insecurity. Values with superscript asterisks * and ** indicate *p* < .05, and *p* < .01, respectively.

Abbreviations: aOR, adjusted odds ratio; CI, confidence interval; RC, Reference category.

^a^ Measured in past 12 months.

^b^ Measured in last 30 days.

^c^ Measured in lifetime.

## 4 DISCUSSION

The current study analyzed a nationally representative dataset comprising of 2,978 adolescents to assess the risk factors of anxiety disorder and the adverse effects of anxiety disorder among adolescent in Bangladesh. We found that the prevalence of anxiety disorders among Bangladeshi adolescents was 4.7%; 4.5% among male adolescent and 5.2% among female adolescent. Several adverse psychosocial factors including feel lonely, being bullied and physically abused were found significant to the increased risk of anxiety disorder. Poor understanding with parents and lack of peer support were also found associated with the increased risk of anxiety disorder. The risk of anxiety disorder further increased with increasing the number of adverse factors. We also found the adolescent experience of anxiety disorder increase their tendency to suicidal ideation, plans, and attempts as well as increase alcohol and drug use. These findings were robust with analyzing nationally representative data and would be helpful for the policymakers to make evidence‐based policy targeting to reduce anxiety among adolescents.

This study has revealed that several adverse psychosocial factors are significant in developing anxiety disorder among adolescent. For instance, a higher likelihood of anxiety disorder was found among lonelier adolescents. Our finding is consistent with the findings of the previous studies that reported increased odds of psychopathological disorders, including anxiety and depressive symptoms among lonelier people (Lasgaard et al., 2011; Stickley et al., 2016). The causes are many, however, the major causes are loneliness affects health behaviors, sleeping quality (Stickley et al., 2016), reduces individuals coping up ability (Drake et al., 2015), and increases adolescents peer victimization behaviors (harassment or bullying) (Acquah et al., 2016). These are the factors that have been found as independent risk factors of anxiety disorder: Another important psychosocial factor, being bullied, has proven to be a statistically significant risk factor for anxiety disorder among adolescent. Bullying is a significant life stressor (Swearer & Hymel, 2015), has negative consequences on adolescent's mental health, low self‐esteem, major depression, etc. and that can extend into adulthood (Juvonen & Graham, 2014). Moreover, Klomek et al. (2015) have demonstrated that childhood bullying victimization can affect three of the most burdensome areas in adulthood: psychopathology, criminality, and suicidality. These study findings also uncovered that adolescent who was physically abused, more likely to be suffered from an anxiety disorder, which does coincide exactly with existing literature (Rehan et al., 2017). These psychosocial factors are vital role players in reducing anxiety disorder in adolescence.

Socio‐environmental factors also develop anxiety disorder among adolescent in different pathways. As found in this study, adolescent having a poor understanding with parents reported higher likelihood anxiety disorder, a finding that was also reported in a study conducted in the USA (Platt et al., 2016). The causes are a lack of family support and parental aversiveness, factors that Cardamone‐Breen et al. (2017) found significantly associated with the anxiety disorder. Moreover, lack of parental support also minimizes the chance of developing adolescent coping up skills (Burstein et al., 2010) and capability of handling adversities (Platt et al., 2016). These increase the risk of developing anxiety disorder among adolescent. Consistent with the available literature, peer supports also found as important predictors of developing anxiety disorder among adolescent (Stapinski et al., 2014). The reason is low peer supports increase adolescent loneliness which contributes to developing anxiety disorder (Pickard et al., 2018). Moreover, peer victimization, which the previous studies found increases the risk of generic and gender‐based risk factors such as, poor life satisfaction, loneliness, etc. also makes adolescent highly vulnerable to develop psychiatric disorders (McLaughlin et al., 2009; Pickering et al., 2019).

In this study, we found a dose‐response relationship between MAE and the likelihood of developing anxiety disorders during adolescence. For example, adolescent experience to increase the number of adverse psychosocial or socio‐environmental factors was found associated with the cumulative increase in developing the risk of anxiety disorder. These findings are in line with previous studies (Bielas et al., 2016; van der Feltz‐Cornelis et al., 2019; Hunt et al., 2017). In addition to the reasons mentioned above, the multiple adverse experiences, either psychosocial or socio‐environmental, have a broad negative impact on a child's development by exhibiting subsequentially changes in adolescents’ emotion and behavior, via disruptions to stress response systems, and also mediate the development of internalizing psychopathology (McLaughlin et al., 2009). Moreover, multiple adverse psychosocial experiences (e.g., loneliness, bullying, etc) together could act as a robust stressor that enhances the susceptibility of developing lower self‐esteem, reduce coping up ability. These are important factors developing anxiety disorder found in several studies (Bielas et al., 2016; Drake et al., 2015; Forbes et al., 2019; Lee & Hankin, 2009). Similar consequences can also occur for multiple socio‐environmental experiences.

We found increased likelihoods of suicidal ideation, plan, and attempts, as well as alcohol and drug use among adolescent who reported anxiety disorder than their counterpart. These findings are in line with the previous studies conducted in Bangladesh and other developing countries (Asante et al., 2017; Conway et al., 2016; Khan et al., 2020; Randall et al., 2014). Furthermore, there is a straight connection with anxiety and impulsivity: anxious individuals are more likely to have impulsive decision‐making tendencies (Xia et al., 2017). Impulsivity accelerates adolescents’ engagement in violent and risk‐taking behaviors, such as substance use (Charles et al., 2016), and suicidal behaviors (McHugh et al., 2019), etc. Researcher highly suggests an evidence‐based primary treatment of mental disorders like anxiety, depression, etc. is essential targeted to youths at risk to prevent suicidal behaviors and substance abuse (Conway et al., 2016; Hegerl, 2016).

There are some strengths and limitations of this study. Firstly, we analyzed GSHS data which is nationally representative and globally recognized. Secondly, we consider complex survey design and sample weights along with appropriate statistical methods that make this study's results are more precise and reasonable for policymaking. Moreover, we adjusted anxiety disorder with several psychosocial and socio‐environmental factors which is another important strength of this study. However, in the GSHS, no data was collected on specific types and levels of anxiety disorders. The survey was cross‐sectional, so the association reported were correlational only, not casual. Moreover, we did not consider many important confounding variables (e.g., region, place of residence, alcohol use, parental conflict, other psychological problem histories, etc.) in the model which was another important limitation of this study.

## 5 CONCLUSIONS

The current study reported around 4.7% of anxiety disorder among adolescent in Bangladesh and it significantly increasing the tendencies of adolescent suicidal ideation, plans, and attempts and alcohol and drug abuse tendencies. This endeavor has contributed to an understanding of the risk factors of developing anxiety among these populations. Several single and multiple adverse psychosocial and socio‐environmental experiences are accelerating the risk. Social and environmental factors need to enhance positively to support adolescents in reducing anxiety disorder. The study findings emphasize the essentiality of early screening and interventions targeted to youths at risk, which might reduce the rate of anxiety disorder in this age cluster. In‐depth and comprehensive research is urgent to extend the understanding of the etiology of anxiety disorder among the adolescent and identify the policies and preventive interventions to reduce the burden effectively.

## CONFLICT OF INTEREST

The authors have no competing interest to declare.

## AUTHOR CONTRIBUTIONS

MMAK conceptualized the study and analyzed the data. MMAK and MNK interpreted the findings and prepared the draft. MNK critically revised the manuscript. All authors read and approved the final manuscript.

## ETHICAL APPROVAL

Ethical approval for the GSHS survey was obtained from the Ministry of Health and Family Welfare, Dhaka, Bangladesh. The WHO conducted this survey with the collaboration of the Center for Disease Control (CDC). They also supported financially and technically. Further, written permission had been obtained from each participating school and from all classroom teachers. The data is available at the webpage of WHO upon request.

### Peer Review

The peer review history for this article is available at https://publons.com/publon/10.1002/brb3.1899.

## Supporting information

Table S1‐S2

## Data Availability

GSHS tools, data are publicly available at WHO or CDC data repositories and can be accessed freely at https://nada.searo.who.int/index.php/catalog/33/download/244 or https://www.cdc.gov/gshs/countries/seasian/bangladesh.htm (Bangladesh). The data are available upon request.
