# Supplementary material for: Effects of psychosocial and socio‐environmental factors on anxiety disorder among adolescents in Bangladesh
Source: Brain Behav. 2020 Oct 21;10(12):e01899. doi: 10.1002/brb3.1899 (PMC7749541; doi:10.1002/brb3.1899)
Supplement: Supplementary file 1 — Table S1‐S2 [file BRB3-10-e01899-s001.docx]

**S-Table 1.** Association between adolescents’ anxiety disorder and adolescent’s adverse experiences in Bangladesh: Global School-Based Health Survey (GSHS), 2014.

| Risk Factors | Anxiety disorder in adolescents | | | | |
| --- | --- | --- | --- | --- | --- |
|  | **Male** | |  | **Female** | |
|  | **aOR** | **95% CI** |  | **aOR^a^** | **95% CI** |
| Adverse Experiences in Adolescence |  | |  |  | |
| Psychosocial risk Factors |  | |  |  | |
| Loneliness |  | |  |  | |
| No^(RC)^ | 1.00 | ….. |  | 1.00 | ….. |
| Yes | 7.89^**^ | (3.88-16.02) |  | 6.78^**^ | (3.79-12.15) |
| Bullied |  |  |  |  |  |
| No^(RC)^ | 1.00 | ….. |  | 1.00 | ….. |
| Yes | 1.47 | (0.71-3.00) |  | 2.43^**^ | (1.45-4.09) |
| No close friends |  |  |  |  |  |
| No^(RC)^ | 1.00 | ….. |  | 1.00 | ….. |
| Yes | 1.71 | (0.61-4.76) |  | 0.48 | (0.18-1.26) |
| Ever sexual intercourse |  | |  |  | |
| No^(RC)^ | 1.00 | ….. |  | 1.00 | ….. |
| Yes | 1.21 | (0.41-3.51) |  | 0.88 | (0.26-2.90) |
| Smoke cigarettes or other tobacco |  | |  |  | |
| No^(RC)^ | 1.00 | ….. |  | 1.00 | ….. |
| Yes | 1.38 | (0.53-3.59) |  | 2.68^*^ | (1.02-7.09) |
| Physically abused |  |  |  |  |  |
| No^(RC)^ | 1.00 | ….. |  | 1.00 | ….. |
| Yes | 2.28 | (0.92-5.63) |  | 2.21^*^ | (1.00-4.95) |
| Socio-environmental factors |  | |  |  | |
| Parental rarely homework check |  |  |  |  |  |
| No^(RC)^ | 1.00 | ….. |  | 1.00 | ….. |
| Yes | 1.67 | (0.79-3.52) |  | 1.35 | (0.77-2.37) |
| Poor understanding with parents |  |  |  |  |  |
| No^(RC)^ | 1.00 | ….. |  | 1.00 | ….. |
| Yes | 1.47 | (0.65-3.31) |  | 1.81 | (0.92-3.56) |
| Poor parental monitoring |  |  |  |  |  |
| No^(RC)^ | 1.00 | ….. |  | 1.00 | ….. |
| Yes | 0.67 | (0.31-1.47) |  | 0.54 | (0.25-1.14) |
| Lack of peer support |  |  |  |  |  |
| No^(RC)^ | 1.00 | ….. |  | 1.00 | ….. |
| Yes | 1.79 | (0.81-3.95) |  | 2.23^*^ | (1.01-4.91) |
| Parental tobacco or drug use |  |  |  |  |  |
| No^(RC)^ | 1.00 | ….. |  | 1.00 | ….. |
| Yes | 1.97 | (0.97-3.98) |  | 1.15 | (0.59-2.27) |
| Truancy |  |  |  |  |  |
| No^(RC)^ | 1.00 | ….. |  | 1.00 | ….. |
| Yes | 1.13 | (0.54-2.34) |  | 1.36 | (0.74-2.54) |

**Note:** ^a^Model was adjusted for individuals’ age, school grade, food insecurity and all the predictors included in this table. Values with superscript asterisks * and ** indicate *p*<0.05, and *p*<0.01, respectively. (RC): Reference category, uOR: unadjusted odds ratio, CI: confidence interval, aOR: adjusted odds ratio.

**S-Table 2.** Influence of multiple adverse experiences (MAE) on adolescents’ anxiety disorder.

| **Multiple Adverse Experiences** | **Anxiety disorder in adolescents** | | | | |
| --- | --- | --- | --- | --- | --- |
|  | **Male** | |  | **Female** | |
|  | **aOR^a^** | **95% CI** |  | **aOR^a^** | **95% CI** |
| **No. of psychosocial risk factors** |  |  |  |  |  |
| 0**^(RC)^** | 1.00 | ….. |  | 1.00 | ….. |
| 1 | 2.59 | (0.55-12.17) |  | 1.99^*^ | (1.02-3.93) |
| 2 | 5.41^*^ | (1.19-24.53) |  | 3.97^**^ | (1.99-7.92) |
| ≥3 | 14.09^**^ | (3.05-64.99) |  | 20.83^**^ | (10.13-42.82) |
| **No. of adverse socio-environmental factors** | |  |  |  |  |
| 0**^(RC)^** | 1.00 | ….. |  | 1.00 | ….. |
| 1 | 0.47 | (0.15-1.45) |  | 2.19^*^ | (1.02-4.68) |
| 2 | 1.03 | (0.43-2.46) |  | 2.57^*^ | (1.19-5.53) |
| ≥3 | - | - |  | 1.83 | (0.89-3.75) |

**Note:** ^a^Models were adjusted for age, school grade, and food insecurity variable. Values with superscript asterisks * and ** indicate *p*<0.05, and *p*<0.01, respectively. (RC): Reference category, uOR: unadjusted odds ratio, CI: confidence interval, aOR: adjusted odds ratio.
